# Texture analysis using proton density and T2 relaxation in patients with histological usual interstitial pneumonia (UIP) or nonspecific interstitial pneumonia (NSIP)

**DOI:** 10.1371/journal.pone.0177689

**Published:** 2017-05-16

**Authors:** Maria T. A. Buzan, Andreas Wetscherek, Claus Peter Heussel, Michael Kreuter, Felix J. Herth, Arne Warth, Hans-Ulrich Kauczor, Carmen Monica Pop, Julien Dinkel

**Affiliations:** 1 Department of Pneumology, Iuliu Hatieganu University of Medicine and Pharmacy, Cluj-Napoca, Romania; 2 Department of Diagnostic and Interventional Radiology with Nuclear Medicine, Thoraxklinik at Heidelberg University Hospital, Heidelberg, Germany; 3 Department of Diagnostic and Interventional Radiology, University Hospital Heidelberg, Heidelberg, Germany; 4 Translational Lung Research Center Heidelberg (TLRC), Member of the German Center for Lung Research (DZL), Heidelberg, Germany; 5 Joint Department of Physics, The Institute of Cancer Research and the Royal Marsden NHS Foundation Trust, London, United Kingdom; 6 Center for Rare and Interstitial Lung Diseases, Pneumology and respiratory critical care medicine, Thoraxklinik, Heidelberg University Hospital, Heidelberg, Germany; 7 Institute for Pathology, University Hospital Heidelberg, Heidelberg, Germany; 8 Institute for Clinical Radiology, Ludwig-Maximilians-University Hospital Munich, Munich, Germany; Universitatsklinikum Freiburg, GERMANY

## Abstract

**Objectives:**

The purpose of our study was to assess proton density (PD) and T2 relaxation time of usual interstitial pneumonia (UIP) and nonspecific interstitial pneumonia (NSIP) and to evaluate their utility in differentiating the two patterns. Furthermore, we aim to investigate whether these two parameters could help differentiate active-inflammatory and stable-fibrotic lesions in NSIP.

**Methods:**

32 patients (mean age: 69 years; M:F, 1:1) with pathologically proven disease (UIP:NSIP, 1:1), underwent thoracic thin-section multislice CT scan and 1.5T MRI. A total of 437 regions-of-interest (ROIs) were classified at CT as advanced, moderate or mild alterations. Based on multi-echo single-shot TSE sequence acquired at five echo times, with breath-holding at end-expiration and ECG-triggering, entire lung T2 and PD maps were generated from each subject. The T2 relaxation time and the respective signal intensity were quantified by performing a ROI measurement on the T2 and PD maps in the corresponding CT selected areas of the lung.

**Results:**

UIP and NSIP regional patterns could not be differentiated by T2 relaxation times or PD values alone. Overall, a strong positive correlation was found between T2 relaxation and PD in NSIP, r = 0.64, p<0.001; however, this correlation was weak in UIP, r = 0.20, p = 0.01. T2 relaxation showed significant statistical difference between active-inflammatory and stable-fibrotic NSIP regions at all levels, p<0.05, while for the analysis of ventral lesions PD proved no statistical difference, p>0.05.

**Conclusions:**

T2 relaxation times and PD values may provide helpful quantitative information for differentiating NSIP from UIP pattern. These parameters have the potential to differentiate active-inflammatory and stable-fibrotic lesions in NSIP.

## Introduction

Interstitial lung diseases comprise a group of diffuse parenchymal pulmonary diseases that are classified together due to their similar clinical, radiologic, physiologic, and/or pathologic manifestations [1]. The new ATS/ERS/JRS/ALAT guidelines for idiopathic pulmonary fibrosis provide criteria to define the usual interstitial pneumonia (UIP) pattern, possible UIP pattern, and patterns inconsistent with UIP on HRCT [2]. Idiopathic nonspecific interstitial pneumonia (NSIP) was accepted as a subtype in 2008 [3], after Katzenstein and Fiorelli introduced the term for those cases of interstitial pneumonia that cannot be pathologically categorized as another type of idiopathic interstitial pneumonia [4]. The radiological patterns of UIP and some NSIP may share criteria for possible UIP pattern. Moreover, a recent review proposed that when a possible UIP pattern is identified, the clinical problem most frequently encountered is to distinguish UIP from NSIP [5].

In idiopathic disease, with the exception that patients displaying NSIP pattern are more commonly female and generally have a younger mean age than those exhibiting UIP pattern [6], there is substantial overlap in the clinical and physiologic features between the two forms, both presenting insidious onset of dyspnea, dry cough and a restrictive ventilatory defect [7].

The recommended therapy and overall prognosis are distinct, however, making accurate diagnosis of this two entities critical [8,9]. Progressive deterioration of lung function and early death are specific for UIP pattern, regardless of its cause (idiopathic or connective tissue related [10])–idiopathic pulmonary fibrosis carries the worst prognosis, with a 5-year survival of approximately 30% [11]. On the other hand, for NSIP stabilization or improvement is more characteristic [12].

Several reports have shown that, in some cases, the differential diagnosis between UIP and NSIP by CT alone is not always straightforward, requiring additional surgical lung biopsy [13–19]. However, lung biopsy carries some risk and morbidity [20,21], therefore the identification of patients with UIP or NSIP by a noninvasive procedure, such as thoracic MRI, would prove to be a valuable clinical resource. Yet, to date and to the best of our knowledge no study has attempted to provide relevant evidence in this matter using MRI techniques.

Moreover, despite its importance for therapy decision-making and for prediction of treatment response, the radiological distinction between active-inflammatory and stable-fibrotic lesions in NSIP remains challenging. The feasibility of measuring lung water content and distribution by MRI techniques has been well recognized since the 1980s [22–25]. However, so far quantitative techniques such as T2 relaxation and PD for inflammatory activity assessment in chronic interstitial lung disease have only been performed on animal models of bleomycin induced fibrosis [26–28]. We aim to investigate for the first time the clinical utility of these methods applied to a cohort of patients with NSIP using a clinical 1.5T MRI scanner.

Giving the histological differences between UIP and NSIP [1], we hypothesized that an analysis using quantitative MRI parameters such as T2 relaxation times and proton density (PD) values may reveal specific features of lung microstructure valuable for the distinction of UIP and NSIP. The purpose of our study was to assess PD and T2 relaxation of UIP and NSIP and to evaluate their utility in differentiating the two patterns. Furthermore, we aim to investigate whether these two parameters could help differentiate active-inflammatory and stable-fibrotic lesions in NSIP.

## Materials and methods

### Patients

This cross-sectional prospective study included 34 patients who displayed an UIP or NSIP pattern, between January 2014 and February 2015. The ethics committee of the University of Heidelberg approved this study (clearance number S-318/2013). Written informed consent was obtained from each patient before performing the study examinations. The inclusion criterion was that the patient had pathologically confirmed UIP or NSIP pattern. All patients underwent thoracic thin-section multislice CT scan and 1.5T MRI. Two patients were excluded because at least one of the two examinations was incomplete. Informed consent was obtained from each patient.

The 32 patients included were 16 males and 16 females, with a mean age of 69 years (range 40–81 years). All diagnoses were made, according to current guidelines, by consensus in a multidisciplinary meeting, after reviewing the clinical findings, results of pulmonary function and laboratory tests, thin-section CT scan and biopsy reports. There were 16 UIP cases (14 idiopathic pulmonary fibrosis and 2 connective tissue disease-related UIP) and 16 NSIP patients (7 idiopathic and 9 with connective tissue disease-related NSIP; 8 with suspected inflammatory activity and 8 with stable disease). The current clinical presentation, pulmonary function tests and thin-section CT scan were used in all cases to categorize patients as having stable disease or suspected inflammatory activity. Stable disease was considered when there was no interval change in clinical presentation, pulmonary function tests and CT appearances from the previous assessment. Patients with suspected inflammatory activity presented with sudden worsening in symptomatology and pulmonary function tests. Possible infection was excluded clinically and the patients underwent chest CT. When ground glass opacities were noted an additional MRI of the lung was performed during the same day (6 cases), the subsequent day (1 case), and the second day after the CT scan (1 case). Twelve patients were previously included in a study analyzing the T2 relaxation time of different CT patterns in interstitial lung disease [29].

### CT imaging

The thin-section MDCT examinations were performed using a 64-detector CT system (Somatom Definition AS, Siemens Medical Systems, Erlangen, Germany), with scans obtained from the lung apex to the diaphragm, during breath-holding at the end of full inspiration. The helical scan protocol applied was: 64×0.6 mm collimation, 1.5 pitch, 0.33 s/rotation, 300–330 mm field of view, 512×512 matrix, 120 kV, 70 mAs. Reconstructions of all thin-section CT images were performed as contiguous slices of 1.0 mm thickness by means of a standard iterative algorithm (I40) and a lung iterative algorithm (I70).

### MR imaging

MRI was performed on a clinical 1.5-T MR whole-body unit (Magnetom Aera, Siemens Medical Systems, Erlangen, Germany). Entire lung T2 sagittal maps were generated based on multi-echo single shot turbo spin echo sequences [TE: 20, 40, 79, 140, 179 ms, each acquisition: 30 slices of 10 mm thickness and 3.125x3.125 mm pixel size (matrix: 104×128)], as described in a previous article [29]. A nonrigid image registration [30], using the first acquisition (TE = 20ms) as reference image, was applied to correct for residual motion mismatch between the volumes at different TE. PD intensity maps, representing the approximation of the MR signal intensity in the limit *TE*→0, were automatically produced from the acquired data set, together with the T2 maps, using the commercial mapping tool pack provided by the MR system post-processing software.

### Image analysis and statistics

One thoracic radiologist (with >5 years of experience) defined the reference images and representative regions of interest (ROIs) while reviewing the CT scans. The ROIs exhibited different degrees of regional parenchymal impairment, according to the density of fibrosis on CT images and presence of parenchymal architectural distortion (lung volume loss, bronchiectasis or scaring), as follows: *advanced* fibrosis with marked architectural distortion (dense fibrosis with severe lung volume loss and traction bronchiectasis, honeycombing or complete scaring), *moderate* disease including reticulation with or without architectural distortion, and *mild* alterations consisting of ground-glass opacities with minimal or no architectural distortion. Honeycombing, reticulation and ground-glass opacities were defined according to the Fleischner Society nomenclature [31]. When a mixture of reticulation and ground glass opacity was present, the region was included in the moderate disease category. The number of measurements per patient was dependent on the extent of the disease and the extent of each regional pattern.

The same radiologist involved in ROI definition quantified the T2 relaxation time on the T2 maps and the respective signal intensities on PD maps, by performing a ROI measurement in the corresponding CT selected areas of the lung. A circular or ovoid ROI was drawn as large as possible to cover an area of >100 mm^2^ and was placed to avoid lesion borders to reduce partial volume-averaging effects. Moreover, the ROI was positioned to avoid large blood vessels and main airways. In order to minimize the clustering effect, only one ROI was selected for a specific type of lesion on each map, or, if the same pattern was extensively present on the same image, the different ROIs were chosen from distinct lung lobes.

Data normalization on the PD measurements was performed to correct for the signal gain variability, using the spleen intensity value as a reference for each patient, since we assumed this organ should have the most constant assessment between patients and none of our patients had a diagnosis of splenic disease. Because the T2 relaxation time of ground-glass opacities and reticulation seems to be different between the two lungs [29] and PD should be affected by gravity, the analysis for each type of lesion was done according to ROI’s topology.

Statistical analysis was performed using R statistical software version 2.15.1 (R Foundation for Statistical Computing, Vienna, Austria). Continuous variables were summarized using median [interquartile range]. The Wilcoxon rank sum test was used to assess the differences between the selected types of lesions. The correlation between the PD intensity values and T2 relaxation times of stable disease was evaluated by Spearman rank correlation test. All probability values were 2-sided, with a level of significance of <0.05.

## Results

The findings in both imaging techniques correlated well on the one-to-one analysis between CT and MR images and ROIs and all 32 patients tolerated well the examination. A total of 437 ROIs, with a median [interquartile] of 13 [10–17] selected sites per patient, were CT classified into advanced (n = 175), moderate (n = 160) and mild (n = 102) lesions. Further details about the number of measurements for each type of regional severity within the overall CT pattern are presented in Tables 1 and 2.

**Table 1:** Median and range of T2 relaxation time (ms) for each regional and general pattern.

Pattern	Location	UIP^c^ Median[IQR]	NSIP_stable_^d^ Median [IQR]	NSIP_active_^e^ Median [IQR]	P-value*{(UIP-NSIP_stable_)/ (NSIP_stable_-NSIP_active_)}
**Mild**	RL^a^	61 [60–63]	61 [59–65]	77 [75–80]	{(0.87)/(**<0.001**)}
	LL^b^	61 [60–66]	74 [65–77]	86 [80–96]	{(0.86)/(**0.04**)}
	ventral		62 [60–69]	71 [69–78]	{/(**<0.01**)}
**Moderate**	RL	77 [73–80]	73 [70–77]	82 [75–91]	{(**<0.01**)/(**<0.0001**)}
	LL	77 [74–79]	81 [77–83]	100 [91–106]	{(0.08)/(**<0.0001**)}
	ventral		74 [71–77]	104 [95–106]	{/(**0.02**)}
**Advanced**		85 [80–91]	82 [79–88]		{(0.05)/}

^a^right lung

^b^left lung

^c^usual interstitial pneumonia (mild, n = 7; moderate, n = 59; advanced, n = 130)

^d^stable nonspecific interstitial pneumonia (mild, n = 24; moderate, n = 45; advanced, n = 45)

^e^active nonspecific interstitial pneumonia (mild, n = 71; moderate, n = 56)

*from Wilcoxon rank sum test

**Table 2:** Median and range of proton density (a.u.) for each regional and general pattern.

Pattern	Location	UIP^a^ Median [IQR]	NSIP_stable_^b^ Median [IQR]	NSIP_active_^c^ Median [IQR]	P-value*{(UIP-NSIP_stable_)/ (NSIP_stable_-NSIP_active_)}
**Mild**	Ventral	52 [48–54]	51 [44–64]	57 [52–62]	{(0.9)/(0.8)}
	Dorsal	35 [32–40]	45 [41–61]	109 [96–126]	{(0.06)/(**<0.0001**)}
**Moderate**	Ventral	57 [54–71]	49 [46–56]	57 [54–67]	{(0.2)/(0.2)}
	Dorsal	89 [65–104]	79 [67–88]	101 [84–113]	{(0.08)/(**<0.0001**)}
**Advanced**	Ventral	57 [49–76]	57 [54–68]		{(0.8)/}
	Dorsal	80 [59–103]	96 [89–109]		{(**<0.0.001**)/}

^a^usual interstitial pneumonia (mild, n = 7; moderate, n = 59; advanced, n = 130)

^b^stable nonspecific interstitial pneumonia (mild, n = 24; moderate, n = 45; advanced, n = 45)

^c^active nonspecific interstitial pneumonia (mild, n = 71; moderate, n = 56)

*from Wilcoxon rank sum test

The same tables show the median and interquartile range of T2 relaxation time and PD respectively, for the three types of lesions according to their localization. No significant statistical difference was found between stable UIP and stable NSIP regarding T2 relaxation times, at any of the analyzed levels, with the exception of moderate disease in the right lung, p<0.01. No significant statistical difference was found between stable UIP and stable NSIP regarding PD, at any of the analyzed levels, with the exception of advanced disease with dorsal localization, where NSIP show significantly higher values as compared to UIP, p<0.001. For dorsal lesions, a strong positive correlation was found between T2 relaxation and PD in stable NSIP, r = 0.64, p<0.001; however, this correlation was weak in stable UIP, r = 0.20, p = 0.01.

**Fig 1:**
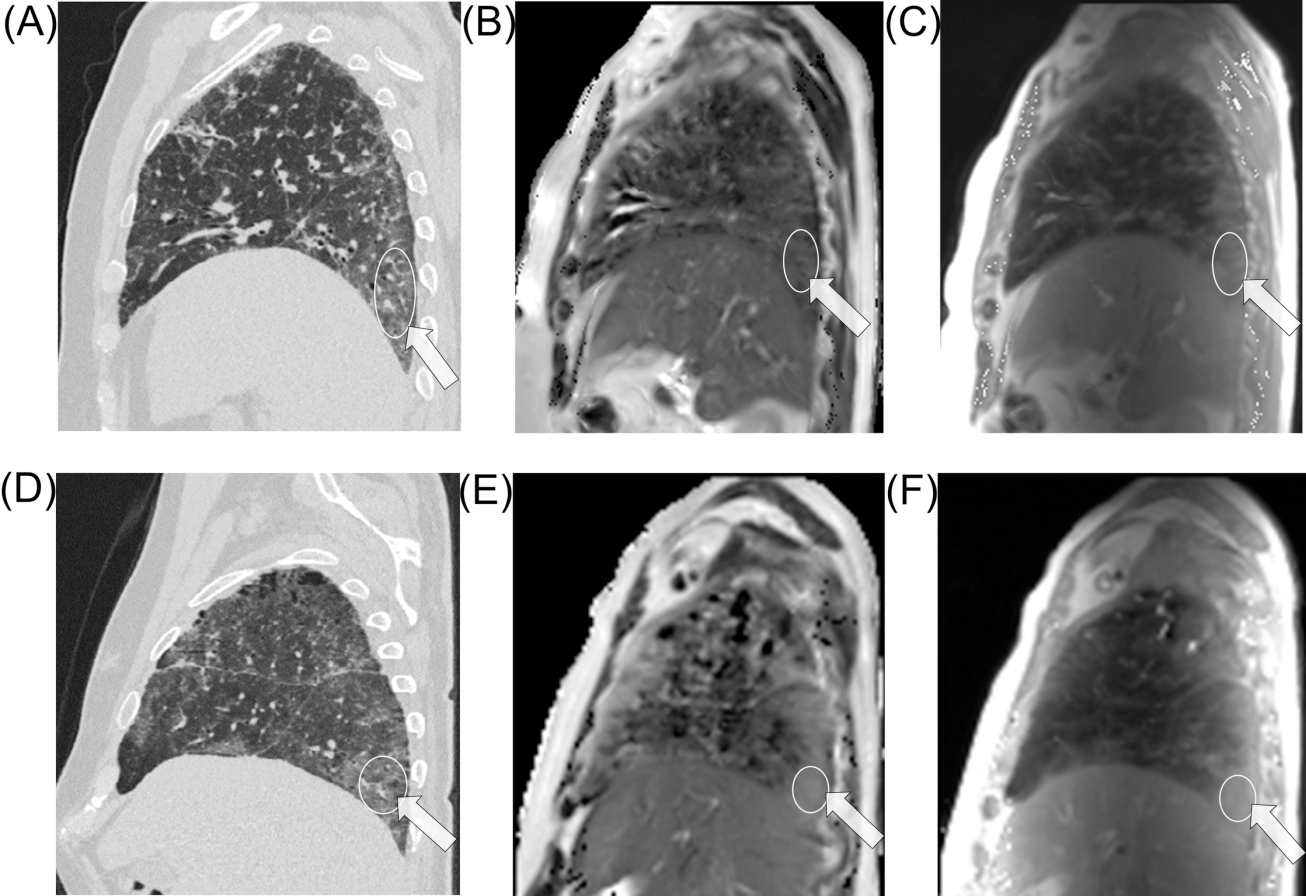
A 59 year old male patient with stable NSIP: image A is the sagittal reformat CT reference image, with the arrow indicating the region of interest, followed by the T2 map, image B and PD intensity map, image C. Below is the case of a 65 year old male patient with NSIP and suspected inflammatory activity and his corresponding images, D to F. Both cases show similar CT morphology and inhomogeneous T2 relaxation and PD of the lung; there is higher T2 relaxation and PD intensity on both type of maps in the active disease case. Exemplary ROI placement in relevant pathological areas is demonstrated.

In NSIP, a strong statistical difference was present between the PD intensity values in active vs. stable disease for both mild and moderate lesions with dorsal localization, p<0.0001, but not for ventral lesions, p>0.05. A significant statistical difference was found between the T2 relaxation time of active-inflammatory vs. stable-fibrotic alterations at all levels, including ventral lesions, p<0.05. Fig 1 shows two CT morphological similar cases for comparison of active-inflammatory and stable-fibrotic mild alterations in NSIP.

## Discussion

In this prospective study, we presented, to the best of our knowledge, the first quantitative MRI description, using T2 relaxation times and PD values, in patients with pathologically proven UIP and NSIP. Furthermore we have investigated for the first time the clinical utility of these parameters for differentiating active-inflammatory from stable-fibrotic lesions, applied on a cohort of patients with NSIP using a clinical 1.5T MRI scanner.

UIP and NSIP regional patterns could not be differentiated by T2 relaxation times or PD intensity measurements individually. Yet, there seems to be a mismatch between the T2 relaxation time and PD values in stable UIP, whereas there appears to be a good correlation between the two in stable NSIP. Moreover, patients with NSIP and suspected inflammatory activity show significantly higher T2 relaxation times and PD values than those with stable disease. However, T2 relaxation seems to be a more reliable parameter since for the analysis of ventral lesions PD proved no statistical difference.

Regarding T2 relaxation time, the only statistically significant difference we found between UIP and NSIP at regional level was for moderate alterations in the right lung. Suzuki et al. reported a significant difference between the supine and prone positions in the right lung at normal breathing of room air, but not for the left lung, in healthy subjects [32]. The authors’ explanation was that the right lung, having a greater volume and weight, may be more influenced by gravity than the left lung. In our study, the higher values in moderate UIP lesions might be due to a greater degree of perivascular fibrosis in NSIP [33], leading to hypoxic vasoconstriction and lower amount of blood in the affected region. However, no such difference was found regarding mild changes. We assume that the mild alterations were insufficient to produce significant blood volume changes in the analyzed regions, therefore not enough T2 relaxation differences.

Regarding PD, we found a statistically significant difference between UIP and NSIP only for advanced lesions with dorsal localization, UIP showing lower values in this case. This might be due to the presence of air-containing honeycombing lesions (micro- and macro-cysts) which decrease the mean proton density in the region.

The histopathologic hallmark of UIP is spatial heterogeneity, which refers to a patchy distribution of dense parenchymal scar alternating with areas of less affected or normal parenchyma [1]. The histologic abnormalities are based on temporal heterogeneity, reflecting different stages in the evolution of fibrosis within the same biopsy specimen [6]. In contrast to UIP, NSIP is characterized by relative spatial homogeneity of parenchymal lung involvement and by temporal homogeneity of the injury (inflammation and/or fibrosis) [1]. Fibrosis may be masked by partial volume averaging effects form healthy or less affected lung parenchyma, resulting in lower averaged PD values. However, this is unlikely in NSIP due to its spatial homogeneity and might have led to the strong positive correlation we found between T2 relaxation and PD in stable NSIP, and the rather weak one in stable UIP. Some of our ROI measurements present outlying values probably due to presence of certain areas inconsistent with the general pattern. Some studies revealed that different patterns of lung injury can be found in different lobes within the same lung or even within the same lobe [5]. We found 9 ROIs with marked outlying values in 3 patients with idiopathic NSIP. While reviewing the data, we observed that the selected ROIs were classified as advanced disease. We believe this finding is consistent with Silva et al. [16], who found that patients with idiopathic NSIP can progress to an UIP pattern at long-term follow-up. Further studies on larger cohorts are needed to further validate our findings, while extending the analysis by including other types of chronic ILD such as hypersensitivity pneumonitis.

The distinction between active-inflammatory and stable-fibrotic lesions is considered a major determinant of treatment response and long-term survival rate. On high-resolution CT, active inflammation and fibrotic lesions may present similar morphology [34]. MRI is generally expected to facilitate differentiation of active-inflammatory and stable-fibrotic lesions, with the advantage of being non-irradiating and noninvasive. The presence of high signal intensity lesions proved to be a useful predictor of treatment response and clinical outcome [35], while subsequent studies showed that regions with active inflammation had prominent enhancement, whereas fibrotic lesions did not [34].

T2 mapping proved useful in the assessment of edema in myocardial infarction and acute myocarditis, and of hypokinetic regions in Takotsubo cardiomyopathy; furthermore, it may improve detection of cardiac sarcoidosis [36]. Lung T2 has been observed to increase in experimental pulmonary edema and has been found to correlate well with gravimetric measurement of lung water content [25]. Furthermore, the T2 relaxation time and proton density are complementary parameters in the assessment of lung water accumulation of different origins [25]. Our study shows that active-inflammatory lesions have high T2 relaxation times and PD values and these parameters could allow for regional quantification of inflammation. However, T2 relaxation seems to be a more reliable parameter. T2 relaxation showed significant statistical difference between active-inflammatory and stable-fibrotic NSIP lesions at all levels, while, for the analysis of ventral lesions, PD proved no statistical difference. Despite that the values of inflammatory lesions overlap with those of advanced fibrosis, the two can be morphologically differentiated on thin-section CT scans. Moreover, giving that fibrotic lesions are less affected by gravity [29], an additional examination in supine may prove valuable in the further differentiation of stable-fibrotic and active-inflammatory lesions, the latter being under the influence of gravity effects due to the free water content.

Our findings suggest that further research on monitoring of these patients, with additional discussion concerning cutoff values, is warranted. Follow-up studies have shown only minor changes on HRCT, at visual assessment, in the first 6 months after diagnosis, but progressive increase in the extent of fibrotic lesions at 1 year or more from initial diagnosis [11]. Recently, we used another approach, by means of both visual assessment and a fully automatic histogram-based quantitative evaluation, to follow-up IPF patients at 1 year interval, and found substantial difference in Hounsfield unit changes of the 40th (representing mostly ground-glass opacities) and of the 80th (representing mostly reticulation) percentiles of density histogram between treated and untreated patients [37]. Giving that T2 relaxation times and PD values are dependent on water content and density of fibrotic tissue, we expect some earlier changes at a local level, leading to increase of the two parameters values with disease progression and decrease with response to treatment. Such quantitative methods may prove useful in future studies concerning follow-up of these patients and assessment of treatment response in clinical trials. Shin et al. [38] showed that a high fibrotic score and a low carbon monoxide diffusing capacity of the lung appear to be significant independent predictive factors of poor survival in patients with fibrotic idiopathic interstitial pneumonia. Our quantitative evaluation shows increased T2 relaxation times both in advanced fibrosis and inflammatory lesions, anticipating possible prognostic implications.

Our study is subject to several limitations. First, the study included only cases with histological UIP or NSIP and this fact might have contributed to selection bias of cases with atypical disease. Still, this setting is more comparable to clinical practice where typical cases can be diagnosed with CT or through a multidisciplinary approach, without the need of biopsy or advanced imaging. Second, we do not have a recent biopsy for the accurate diagnosis of inflammatory activity. Such a procedure could not be justified in patients who previously underwent a histological diagnosis of their disease due to the increased risk of exacerbation. Third, we chose several ROIs from a single patient in order to have as many measurements for each type of lesion as possible. On the other hand this lead to each patient contributing different numbers of ROIs within the mild, moderate or severe lesion groups. These facts may cause clustering effects that affect the results. However, we adjusted our analysis accordingly and the overall large number of ROIs included (n = 437) should minimize this possible source of error. Moreover, the severity of lesions was very broad, particularly in NSIP stable patients, and patients with honeycombing contributed not only for severe lesions but also for mild or moderate lesion if reticulation or ground glass opacities were present in a slice. Another limitation is that comorbidities, which are frequent in idiopathic pulmonary fibrosis (e.g. pulmonary hypertension) [39], could have influenced the findings. In the present study, we aimed to find differences between UIP and NSIP pattern, and between active-inflammatory and stable-fibrotic lesions, respectively. Further analysis will be needed on larger patient cohorts to assess possible effects of comorbidities that might influence the results.

## Conclusions

The correlation between T2 relaxation times and PD values may prove helpful in differentiating NSIP from UIP pattern, thus decrease the amount of pathologically confirmed NSIP or UIP pattern. The described quantitative MRI methods have the potential to differentiate active-inflammatory and stable-fibrotic lesions in NSIP, therefore possibly allowing for a better stratification of patients who might benefit from immunomodulatory therapy.
